# Effect of polyethylene glycol 4000 supplementation on the performance of yearling male Pedi goats fed dietary mixture levels of *Acacia karroo* leaf meal and *Setaria verticillata* grass hay

**DOI:** 10.1007/s11250-017-1305-9

**Published:** 2017-05-09

**Authors:** David Brown, Jones W. Ng’ambi

**Affiliations:** Department of Agricultural Economics and Animal Production, Faculty of Science and Agriculture, University of Limpopo, Private Bag X1106, Sovenga, South Africa

**Keywords:** *Acacia karroo*, Tannins, Polyethylene glycol, Goat

## Abstract

Eighteen yearling male Pedi goats weighing 21.7 ± 3.1 kg were used in a 42-day trial in a 2 (*Acacia karroo* leaf meal levels) × 3 (levels of PEG 4000) factorial arrangement in a completely randomized design to determine PEG 4000 supplementation levels for optimal productivity of indigenous Pedi goats fed different mixture levels of *A. karroo* leaf meal and *Setaria verticillata* (L.) P.Beauv. grass hay. Each goat was supplemented with 0, 23 or 30 g of PEG 4000 per day in addition to dietary mixture of *A*. *karroo* and *S*. *verticillata* hay. Polyethylene glycol 4000 supplementation had no effect (*P* > 0.05) on nutrient intake of goats. However, a diet × PEG (*P* < 0.05) was observed for intake of all nutrients studied. Dry matter, OM, NDF and ADF intakes per goat were optimized at PEG 4000 supplementation levels of 19.62, 19.62, 19.61 and 19.53 g/goat/day, respectively, for diets containing 20% *A*. *karroo* leaf meal. Polyethylene glycol 4000 supplementation had no effect (*P* > 0.05) on the apparent digestibility of all nutrients. The dietary inclusion level of *A*. *karroo* leaf meal at 20% improved (*P* < 0.05) DM, OM, CP, NDF and ADF digestibility of goats. Crude protein digestibility was optimized at a PEG 4000 supplementation level of 15.78 g/goat/day. Dietary mixture level and PEG 4000 supplementation had no effect (*P* > 0.05) on final weights of Pedi goats. Similar results were observed for blood urea and glucose concentrations of yearling male Pedi goats. However, daily body weight gain was higher (*P* < 0.05) in goats fed 50% *A*. *karroo* leaf meal than those on 20% inclusion level. Polyethylene glycol 4000 has potential to improve the feeding value of tanninifeorus *A*. *karroo* leaf meal.

## Introduction

Goat production contributes immensely to the economy and food security of many smallholder farmers, particularly in the rural areas (Ngambi et al. 2013). Indigenous Pedi goats are important domestic animals in Limpopo Province of South Africa. The breed derived its name from the Bapedi people in the north of the country (Dagris 2007). Pedi goats have small to medium frame with short horns. Coat colour varies from uniform brown to white with a variety of black and white patterns (Snyman 2014). They provide immediate benefits in the form of milk and meat, hide and skins and cash to the poor in the communal areas of South Africa (Ngambi et al. 2013). However, their productivity is constrained by a shortage of good quality feed, especially during the long dry season (Matlebyane et al. 2010). Available fodder during this period is fibrous and low in protein. Poor nutrition results in poor productivity. Undernourished animals are also predisposed to diseases and parasites and may die in extreme cases (Matlebyane et al. 2010).

Browse plants have the potential to alleviate feed shortages and nutritional deficiencies experienced during the critical dry period of the year (Belachew et al. 2013). Indigenous legume trees such as *Colophospermum mopane*, *Brachystegia spiciformis* and *Acacia* genus are gaining importance as protein sources for livestock during the dry season (Dube 2000; Mapiye et al. 2011). Of particular interest to animal nutritionists in Southern Africa is *Acacia karroo* Hayne. *A*. *karroo* is widely spread and abundantly available during the dry season (Brown et al. 2016). The foliage contains high concentrations of crude protein (100–250 g/kg DM) and minerals (Mokoboki et al. 2005) and has the potential to increase productivity of goats feeding on low-quality roughages. However, their use as protein supplement is restricted by the presence of tannins in their foliage. According to Mokoboki et al. (2005), *A*. *karroo* contained 80.7 g/kg DM extracted condensed tannins (CT). At a level above 50 g/kg DM, CT restricts nutrient utilization and digestibility and may bind to digestive enzymes, thus reducing their activities (Christopher 2012). Recommendations on the use of *A*. *karroo* are complicated due to the presence of CT in the plant. *A*. *karroo* leaf meal inclusion levels of 20 and 50% were used in the present study. These inclusions include low and high levels as indicated in the literature (Brown et al. 2016, 2017).

Tannin-binding agents such as polyethylene glycol (PEG) offer a viable technique to enhance the nutritive value of tannin-rich trees and shrubs (Singh et al. 2005; Nsahlai et al. 2011). Polyethylene glycol is an inert unabsorbed molecule that can form stable complexes with tannins, preventing the binding between tannins and proteins and can even displace protein from a pre-formed tannin-protein complex (Makkar 2003; Frutos et al. 2004). Polyethylene glycol 4000 has been used to alleviate negative effects of CT, consequently improving the performance of animals on tanniniferous-based diets (Decandia et al. 2000; Motubatse et al. 2008). However, other authors have found that PEG 4000 supplementation does not improve the performance of animals on tannin-rich diets (Barry et al. 2001; Bhatta et al. 2002). Responses to PEG 4000 supplementation may depend on the acacia species (amount and type of tannins present) and animal breed (Motubatse et al. 2008). In previous experiments, PEG levels of 0, 23 or 30 g were used to alleviate the effects of tannins in ruminants (Silanikove et al. 1996a, b; Motubatse et al. 2008). It is, therefore, important to ascertain the effects of PEG 4000 on the performance of indigenous Pedi goat breeds fed on diets supplemented with tanniniferous *A*. *karroo* leaf meal.

The objective of this study was, therefore, to determine PEG 4000 supplementation levels for optimal productivity of indigenous Pedi goats fed different mixture diets of *A*. *karroo* leaf meal and *Setaria verticillata* (L.) P.Beauv. grass hay. It was hypothesized that PEG 4000 supplementation levels do not have effect on the optimal productivity of indigenous Pedi goats fed different mixture diets of *A*. *karroo* leaf meal and *S*. *verticillata* (L.) P.Beauv. grass hay.

## Materials and methods

### Study site

The study was conducted at the University of Limpopo Experimental farm, South Africa (latitude 27.55° S and longitude 24.77° E) in October 2014. The ambient temperatures at the study site range between 20 and 36 °C during summer and between 5 and 25 °C during winter. Mean annual rainfall is 446.8 mm with the dry season occurring between April and October and the rainy season occurring between November and March.

### Collection, drying and storage of plant material

*A*. *karroo* leaves were harvested during the winter months (June–August). Branches of each shrub were removed manually and placed in a shed. Leaves were allowed to air dry on the branches and then removed by carefully beating the branches with sticks. The leaves were stored in air-tight bags until feeding time. *S. verticillata* (L.) P.Beauv. is a perennial grass belonging to the tribe Paniceae and is widely grown by commercial farmers in Limpopo Province of South Africa. The grass is well grazed during summer and is suitable for hay making. Botanical authentication of the plant materials was done at the Larry Leach Herbarium, Department of Biodiversity, University of Limpopo.

### Animals, management, diet and experimental design

All procedures involving animals were approved by the Animal Research Ethics Committee of the University of Limpopo, South Africa. Eighteen yearling male indigenous Pedi goats with a mean live weight of 21.7 ± 3.1 kg were randomly allocated to six treatments in a 2 (*A. karroo* leaf meal levels) × 3 (levels of PEG 4000) factorial arrangement (SAS 2010), in a completely randomized design. Each treatment had three replicates with one goat per replicate. The six treatments are given in Table 1. Yearling male Pedi goats were selected because they are the ones fattened for meat in the province. The grass and *A*. *karroo* leaves were chopped and thoroughly mixed to avoid diet selection by the animals when fed. The goats were fed ad libitum, allowing a 15% refusal of each diet, as suggested by Kaitho et al. (1996) and the experiment lasted for 42 days. The animals were housed in individual metabolic cages that allowed the separation of faeces and urine (5.5′ × 2.0′) and were given the experimental diets during the study period. Feed intake was recorded on a daily basis during the collection period. Intake was calculated by subtracting leftovers from the feed given. Water was offered ad libitum. The PEG 4000 was dissolved in water (23 or 30 g of PEG 4000/25 ml of water per animal per day), and the contents were given to the animal through dosing daily at 08 h00 before morning feeding (Decandia et al. 2000). Animals not receiving PEG 4000 were dosed with 25 ml of water. The goats were weighed three times, at the start of the experiment, on day 35 and on the 42nd day when data collection ended. Average daily gains were calculated as differences between final and initial body weights divided by number of days of feeding. The weighing of the goats was carried out before morning feeding to avoid feed effect (Sarwatt et al. 2003). During the collection period, daily output of faeces for each animal were collected, mixed and recorded. Ten percent of the faeces were sub-sampled and kept. All the sub-sampled faeces were pooled on animal basis, dried and kept until required for chemical analysis. Blood samples were taken from each goat via the jugular vein before the morning feeding on day 36 and on the last day of the experiment. The blood samples were collected into anticoagulant free bottles, allowed to coagulate at room temperature and centrifuged at 1500×*g* for 10 min. The supernatant sera were then harvested and stored at −20 °C for subsequent biochemical analysis (Olafadehan et al. 2014).

**Table 1:** Feed composition of the experimental diets

	Diets		
Diet code	*Acacia karroo* (%)	*Setaria verticillata* grass (%)	Peg (g/goat/day)
A_20_S_80_P_0_	20	80	0
A_20_S_80_P_23_	20	80	23
A_20_S_80_P_30_	20	80	30
A_50_S_50_P_0_	50	50	0
A_50_S_50_P_23_	50	50	23
A_50_S_50_P_30_	50	50	30

Nutrient composition of *A*. *karroo* and *S*. *verticillata* grass are presented in Table 2. *A*. *karroo* contained 127 g CP/kg DM and 921 g OM/kg DM. It also contained phenolic compounds. *S*. *verticillata* hay contained 79, 779 and 507 g of CP, NDF and ADF per kilogram DM, respectively. *S*. *verticillata* hay contained no traces of phenolic compounds.

**Table 2:** Nutrient composition of *Acacia karroo* leaf meal and *Setaria verticillata* hay

Nutrient	*Acacia karroo*	*Setaria verticillata* hay
Dry matter (g/kg)	971	962
Organic matter (g/kg DM)	921	914
Crude protein (g/kg DM)	127	79
Acid detergent fibre (g/kg DM)	325	507
Neutral detergent fibre (g/kg DM)	380	779
Condensed tannins (% DM)^a^	2.0	0
Total phenolics (% DM)^b^	1.9	0

^a^Condensed tannins as percentage DM leucocyanidin equivalent

^b^Expressed as tannic acid equivalent (%)

The nutritive values of dietary mixtures of *A*. *karroo* and *S*. *verticillata* hay are presented in Table 3. Diet containing 50% *A*. *karroo* leaf meal inclusion level contained 103 g CP/kg DM. The condensed tannin and total phenolic contents were 1.0 and 0.9%, respectively. A diet containing 20% *A*. *karroo* leaf meal inclusion level had 89 g CP/kg DM, 0.4% condensed tannin and 0.3% total phenolic content.

**Table 3:** Chemical composition of the dietary mixtures of *Acacia karroo* leaf meal and *Setaria verticillata* hay

	Diet	
Nutrient	S_80_A_20_	S_50_A_50_
Dry matter (g/kg)	952	970
Organic matter (g/kg DM)	915	917
Crude protein (g/kg DM)	89	103
Acid detergent fibre (g/kg DM)	470	415
Neutral detergent fibre (g/kg DM)	699	579
Condensed tannins (% DM)^a^	0.4	1.0
Total Phenolics (% DM)^b^	0.3	0.9

^a^Condensed tannins as percentage DM leucocyanidin equivalent

^b^Expressed as tannic acid equivalent (%)

### Chemical analyses

Dry matter, organic matter and crude protein were determined using the methods described by AOAC (2005). Neutral detergent fibre (NDF) and acid detergent fibre (ADF) were determined by the method of Van Soest (1994). Total phenolics were determined using Folin-Ciocalteau methods and expressed as tannic acid equivalent (% DM) (Water and Mole 1994). Condensed tannins were determined using the Butanol-HCl method and expressed as leucocyanidin equivalent (% DM) (Porter et al. 1986). Serum glucose was done on a 600: UniCel DxC General Chemistry Analyzer. Serum urea was obtained by the method of Valley et al. (1980).

### Statistical analysis

The effects of PEG 4000 supplementation, level of *A*. *karroo* leaf meal inclusion and their interaction on intake, in vivo digestibility, growth rate and blood metabolic profiles of goats were analyzed using GLM procedures of SAS (2010). Where significant treatment effects were detected, means were separated by Fisher’s least significant difference. The responses in optimal intake, digestibility and body weight change to the level of browse inclusion were modelled using the following quadratic equation:

$$ Y= a+{b}_1 x+{b}_2{x}^2 $$

where *Y* is the feed intake (g/day), digestibility (coefficient) or body weight change (g/day); *a* is the intercept; *b*_1_ and *b*_2_ are the coefficients of the quadratic equation; *x* is the level of inclusion and −*b*_1_/*2b*_*2*_ = level of inclusion value for optimal response. The quadratic model was used because it gave the best fit.

## Results

Results of the effect of PEG 4000 supplementation, *A*. *karroo* leaf meal inclusions and their interaction on diet intake, digestibility, body weight change and blood serum chemistry of yearling male indigenous Pedi goats fed a basal diet of *S*. *verticillata* grass hay are presented in Table 4. Polyethylene glycol 4000 supplementation had no effect (*P* > 0.05) on nutrient intake of goats. However, the interaction effect of diet and PEG was detected (*P* < 0.05) for intake of all nutrients studied. Dry matter, OM, NDF and ADF intakes per goat were optimized at PEG 4000 supplementation levels of 19.62, 19.62, 19.61 and 19.53 g/goat/day, respectively, for diets with a mixture of 20% *A*. *karroo* and 80% *S*. *verticillata* grass hay (Table 5). Dietary mixture level and PEG 4000 supplementation did not affect (*P* > 0.05) daily CP intake of goats.

**Table 4:** Effects of polyethylene glycol 4000 supplementation and *Acacia karroo* leaf meal inclusion on diet intake, digestibility, body weight change and blood serum chemistry of yearling male indigenous Pedi goats fed a *Setaria verticillata* grass hay-based diet

	Peg level (g/goat/day)			SE	^c^Diet level		SE	*P* Value		
	0	23	30		20	50		Peg	Diet	Peg ^c^Diet
Intake (g/goat/day)										
DM	543	580	590	70.40	652^a^	490^b^	57.48	0.8389	0.0338	0.0418
OM	498	531	541	64.48	596^a^	450^b^	52.65	0.8398	0.0350	0.0417
CP	52	54	56	6.72	58	51	5.49	0.9069	0.2770	0.0418
NDF	344	381	383	45.97	455^a^	284^b^	37.53	0.7217	0.0022	0.0439
ADF	239	262	264	31.60	306^a^	204^b^	25.80	0.7616	0.0054	0.0426
Intake (g/kg W^−0.75^)										
DM	49	55	56	4.888	61^a^	45^b^	3.991	0.5742	0.0139	0.1021
OM	45	51	51	4.471	56^a^	41^b^	3.651	0.5749	0.0147	0.1037
CP	4.7	5.2	5.3	0.462	5.5	4.7	0.378	0.6537	0.1654	0.1060
NDF	31	36	36	3.246	43^a^	26^b^	2.651	0.4912	0.0008	0.1033
ADF	21	25	25	2.214	29^a^	19^b^	1.807	0.5057	0.0019	0.0994
Digestibility (decimal)										
DM	0.71	0.72	0.73	0.007	0.74^a^	0.71^b^	0.005	0.1002	0.0024	0.1491
OM	0.73	0.72	0.74	0.008	0.75^a^	0.71^b^	0.007	0.4066	0.0056	0.0604
CP	0.62	0.63	0.64	0.016	0.68^a^	0.59^b^	0.013	0.7355	0.0004	0.0902
NDF	0.64	0.66	0.67	0.009	0.71^a^	0.60^b^	0.007	0.1104	<.0001	0.3816
ADF	0.63	0.65	0.69	0.030	0.70^a^	0.62^b^	0.025	0.3464	0.0365	0.7346
Initial BW (kg)	23.8	22.5	22.2	1.095	22.7	23.0	0.894	0.5660	0.7769	0.0666
Final BW (kg)	24.1	22.8	23.0	1.150	22.9	23.7	0.939	0.6765	0.5800	0.0760
Gain (g/goat/day)	44.8	74.6	72.1	18.00	36.1^b^	91.6^a^	14.69	0.4534	0.0203	0.3242
Urea (mmol/L)	2.9	3.0	2.5	0.318	2.7	2.8	0.260	0.5131	0.6589	0.5515
Glucose (mmol/L)	2.7	2.8	2.9	0.058	2.8	2.8	0.260	0.1744	0.6308	0.5023

*SE* standard error, *BW* body weight

^a, b^Means with different superscripts within rows are significantly different (*P* < 0.05); SE: Standard error; BW: Body weight

^c^Diet level refers to the percentage of inclusion of *Acacia karroo* leaf meal

**Table 5:** PEG 4000 supplementation levels for optimal dietary intakes in indigenous Pedi goats fed diets with a mixture of 20% *Acacia karroo* leaf meal and 80% *Setaria verticillata* grass hay

Variable	Formula	PEG Supplementation level	Optimal Y-Level	*r* ^2^
Intake (g/goat/day)				
DM	*Y* = 495 + 28.482× + −0.726 × ^2^	19.62	774.75	0.95
OM	*Y* = 452.99 + 26.058× + −0.664 × ^2^	19.62	708.67	0.94
NDF	*Y* = 346 + 19.884× + −0.507 × ^2^	19.61	540.95	0.92
ADF	*Y* = 233 + 13.555× + −0.347 × ^2^	19.53	365.36	0.91
Intake (g/kgW^−0.75^)				
DM	*Y* = 49 + 2.237× + −0.055 × ^2^	20.34	71.75	0.85
OM	*Y* = 45 + 2.083× + −0.052 × ^2^	20.03	65.86	0.89
NDF	*Y* = 34 + 1.480× + −0.036 × ^2^	20.56	49.21	0.84
ADF	*Y* = 23 + 0.987× + −0.024 × ^2^	20.56	33.14	0.78
Digestibility (decimal)				
CP	*Y* = 0.660 + 0.0082× + −0.0002 × ^2^	15.78	0.72	0.93

*r* ^2^ coefficient of determination

Similarly, goats fed a dietary mixture of 20% *A*. *karroo* leaf meal and 80% *S*. *verticillata* grass hay had higher (*P* < 0.05) DM, OM, NDF and ADF intakes per metabolic weight than those on 50% *A*. *karroo* leaf meal and 50% *S*. *verticillata* grass hay (Table 4). Dry matter, OM, NDF and ADF intakes per goat metabolic weight were optimized at PEG 4000 supplementation levels of 20.34, 20.03, 20.56 and 20.56 g/goat/day, respectively, for goats on a mixture of 20% *A*. *karroo* leaf meal and 80% *S*. *verticillata* grass hay (Table 5). Dietary mixture level and PEG 4000 supplementation did not affect (*P* > 0.05) daily CP intake per metabolic weight of goats.

Polyethylene glycol 4000 supplementation had no effect (*P* > 0.05) on the apparent digestibility of all nutrients. The dietary inclusion level of *A*. *karroo* leaf meal at 20% improved (*P* < 0.05) DM, OM, CP, NDF and ADF digestibility of goats. Crude protein digestibility was optimized at a PEG 4000 supplementation level of 15.78 g/goat/day (Table 5).

Dietary mixture level and PEG 4000 supplementation had no effect (*P* > 0.05) on final weights of Pedi goats. Similar results were observed for blood urea and glucose concentrations of yearling male Pedi goats. However, daily body weight gain was higher (*P* < 0.05) in goats fed 50% *A*. *karroo* leaf meal than those on 20% inclusion level.

## Discussion

*A*. *karroo* leaf meal contained 127 g CP per kg DM, indicating that it has potential as a browse source for goats. Leaves of fodder trees have been used as supplements to grass hay-based diets, especially during the critical dry periods of the year (Tshabalala et al. 2013). These tree fodders have been incorporated into the rations of ruminants as substitutes for more expensive processed protein sources (Norton 1994). The condensed tannin contents of the dietary mixture of *A*. *karroo* leaf meal and *S*. *verticillata* hay varied from 4.0 g/kg DM for diet containing a 20% *A*. *karroo* inclusion level to 10.0 g/kg DM for the diet containing 50% *A*. *karroo.* Dube et al. (2001) and Mokoboki et al. (2005) indicated that *A*. *karroo* leaves contain high levels of extracted condensed tannins (CT), ranging from 55 to 110 g/kg DM.

In the present study, PEG by diet interaction increased the intake of all nutrients. Dry matter, OM, NDF and ADF intakes were optimized at PEG 4000 supplementation levels of 19.62, 19.62, 19.61 and 19.53 g/goat, respectively. This finding is similar to those of Yisehak et al. (2013) who reported significant interaction effect of diet and PEG on DM, OM, CP, NDF and ADF. The considerable improvement in the intake of all studied nutrients after the incorporation of PEG reveals the positive impact of PEG when added to tanniniferous diet (Frutos et al. 2004). According to Makkar (2003), intake of tanniniferous diet can be increased by using compound that has high affinity for tannins like PEG. Silanikove et al. (1996a, b) and Titus et al. (2001) also reported that PEG supplementation increased intake of tanniniferous browse foliage. The incorporation of PEG in the diet breaks tannin-fibre complexes, which consequently allows their digestion by microbial enzymes (Frutos et al. 2004; Yisehak et al. 2013). Inactivation of tannins through PEG increased availability of nutrients (protein, carbohydrates and minerals) to the animal and decreased microbial inhibition leading to higher animal performance (Makkar 2003).

Polyethylene glycol supplementation had no effect on the apparent digestibility of nutrients in the present study. Silanikove et al. (1996a, b) found that goats fed high-tannin *Pistacia lentiscus* with PEG (10 g/day) had negative CP digestibility. Similarly, Motubatse et al. (2008) reported that PEG 4000 supplementation did not affect NDF and ADF digestibility in Pedi goats fed *Acacia nilotica* leaf meal. Contrary to this result, other authors have reported significant improvement in the apparent digestibility of nutrients (OM and CP) with PEG supplementation (Barry et al. 1986; Ben Salem et al. 2005; Yisehak et al. 2013). The effect of PEG depends on the level of protein in the diet. The higher the level of protein in a diet, the lower is the effect of PEG (Austin et al. 1989; Makkar and Becker 1996; Alonso-Diaz et al. 2010). This may account for the reduced nutrient digestibility observed for goats on 50% *A*. *karroo* leaf meal.

The final body weight of goats was not improved with dietary mixture level and PEG supplementation. These findings are similar to those of Ben Salem et al. (2005), who reported non-significant effects in the final body weight of goats fed tannin-rich oak (*Quercus coccifera* L.) and supplemented with PEG 4000. In contrast, Motubatse et al. (2008) reported improved final body weights in Pedi goats fed different levels of *A*. *nilotica* leaf meal and supplemented with PEG 4000. The difference in responses may be related to differences in the chemical composition of the browse species (Madibela et al. 2006). It should be noted that discrepancies in body weight change may also be due to changes in rumen fill, which might not represent changes occurring in the body tissues (Motubatse et al. 2008). *A*. *karroo* leaf meal at 50% improved daily body weight gain of goats. This observation may be due to higher protein content in the diet. Improvement in weight gain of goats on tanniniferous diets have been document (Solaiman et al. 2010; Gwanzura et al. 2011; Brown et al. 2017). Rumen microorganisms are able to metabolize tannins or remain active in a high tannin environment and overcome their detrimental effects, which in turn improves animal performance (Makkar 2003).

There was no consistent effect of PEG 4000 supplementation and dietary treatments on serum urea and glucose concentration of the goats. These findings are similar to the report of Ben Salem et al. (2005). Serum glucose was within the normal range of 2.78–4.16 mmol/l reported for healthy goats (Kaneko 1997). Serum urea level was below the normal established range of 3.5–10.7 mmol/l (Sirois 1995). The reason for depressed serum urea in the present experiment merits further study.

In conclusion, *A*. *karroo* leaf meal has potential as a protein feed for ruminants. However, it also contained relatively high amounts of condensed tannins, which can deter intake and digestion in goats. The negative effects of tannins on nutrient intake could be reversed by supplementing tannin-binding agents such as PEG. Supplementation level of about 20 g of PEG 4000 per goat per day optimized DM, OM, NDF and ADF intakes. This level of supplementation is recommended if intake is the parameter of interest. Crude protein digestibility was optimized at a PEG 4000 supplementation level of 15.78 g per goat per day. This supplementation level for optimal CP digestibility is lower than the 20% for intake. Thus, the supplementation level for optimal productivity depended on the production parameters of interest. This has implications on supplementation recommendations. Dietary inclusion level of *A*. *karroo* at 50% did not improve intake and digestibility of the animals. It is possible that PEG 4000 was not enough to have effect on the performance of the goats. Similarly, the condensed tannins may have exerted adverse effects on the performance of the goats. All this requires further studies.
